# Vascular Mimicry: The Next Big Glioblastoma Target

**DOI:** 10.4172/2168-9652.1000e140

**Published:** 2015-08-31

**Authors:** Ali S Arbab, Meenu Jain, BR Achyut

**Affiliations:** Tumor Angiogenesis Lab, Biochemistry and Molecular Biology Department, Cancer Center, Georgia Regents University, Augusta, GA 30912, USA

Glioblastoma (GBM), a grade IV glioma classified by World Health Organization (WHO), is considered highly malignant, vascular and invasive subtype [1]. GBM is most lethal during first year after initial diagnosis despite surgical resection, radiotherapy and/or chemotherapy [1,2]. Median survival of patients diagnosed with GBM is only 12 to 15 months [1,2]. Anti-angiogenic therapies (AAT) were used as an adjuvant mainly against vascular endothelial growth factor and its receptors (VEGF-VEGFRs) to normalize tumor vasculatures in GBM patients. However, all of them provided minimal to none effect with no change in overall survival [3]. Hypoxia and neovascularization are histopathologic features of GBM [4]. Hypoxia activated proangiogenic, invasion and metastasis associated gene signatures, enabling tumor to become more angiogenic, invasive and malignant in a compromised microenvironment [5,6]. GBM tumor vessels are tortuous, disorganized, highly permeable, and have abnormal endothelial cells (ECs), pericyte coverage, and basement membrane structure [7,8]. Conventionally, tumor vessel formation occurs through angiogenesis, which is mediated by proliferation and migration of resident ECs [9]. Instead, vasculogenesis originates from circulating bone marrow derived cells (BMDCs) or endothelial progenitor cells (EPCs), which express VEGFR2, are recruited by VEGF followed by differentiation and incorporation into new tumor blood vessels [10].

So far, published studies have focused on the endothelial cell-associated tumor vasculature development and BMDCs mediated vasculogenic mechanisms in tumor development. However, depending on tumor development, neovascularization occurs by alternate mechanisms such as vascular mimicry (VM) [11,12] and vascular trans differentiation from glioma stem cells (GSCs) [13-15]. Recently, VM has been given much attention, which is a process on neovascularization in tumor development [16,17]. VM channels are lined exclusively by tumor cells mimicking the function of endothelial cells. VM channels connect with endothelium-dependent vessels to create a network that provides for the tumor's growth, invasion and metastasis [18]. Investigation of molecular mechanism of VM channel formation is poorly studied as well as VEGF receptors are expressed at high level by GBM tumor cells and contribution of VM in the neovascularization is poorly studied in GBM.

Newly-formed vessels in GBM are thought to arise by sprouting of pre-existing brain capillaries [19]. VM is a tumor cell-constituted, matrix-embedded fluid-conducting meshwork that is independent of endothelial cells and is positively correlated with poor prognosis [19,20]. Number of glioma polyploid giant cancer cells (PGCCs) associated with VM formation and tumor grade in human glioma [21]. Tree kinds of microcirculation pattern existed in human glioma including VM, mosaic vessel (MV) and endothelium dependent vessel. There were more VM and MVs in high grade gliomas than those in low grade gliomas [21]. Authors reported that vascular channels of VM in GBM were composed of mural-like tumor cells that strongly express VEGFR2 [11,12]. GBM cell lines U87 and patient derived glioma cells, both of which express VEGFR2 and exhibit a vascular phenotype on matrigel [11]. VEGFR2 is an essential molecule to sustain the “stemness” of glioma stem cell-like cells (GSLCs) and their capacity to initiate tumor vasculature and growth [22]. GLSCs trans-differentiated into mural cells to drive VM in GBM. Most of them consisted of blood-perfused vascular channels that coexpress mural cell markers αSMA and PDGFRβ, EGFR, and VEGFR2, but not CD31 or VE-cadherin [12]. This microvasculature coexisted with endothelial cell-associated vessels. vasculogenic capacity of CD133+ brain GLSCs and their cellular plasticity contribute to form vessel-like structures and provide a blood supply to GBM cells [14,15,20,23]. In addition, glioma cells mimic endothelial cells and incorporate into tumor vasculature, which may contribute to radio-resistance observed in GBM [24].

Like other hallmarks of inflammation such as production of ROS and DNA damage etc., VM may be the product of cancer associated inflammation. This is evident by several previous reports. For example, IL8 is identified as a signature paracrine cytokine, and blockade of IL8 but not VEGF prevented vasculature development [25]. Extracellular IL8 trans-activated VEGFR2 and induces phosphorylation of extracellular signal-regulated kinases [25]. Since cancer stem cells (CSCs) are involved in the aggressive behavior of tumor, ubiquitin-specific protease 44 (USP44) positive CSCs subclones under inflammatory environment showed increased levels of IL6 and IL8 (ALDH1+/USP44+/IL-6+/IL-8+) that may contribute to the prediction of VM formation and invasiveness of tumor [26]. Tumor cell derived TNFα constitutes a TME signal that promoted endothelial phenotype via upregulation of the fibronectin receptor α(5)β(1) through trans-differentiation [27]. TNFα-treated monocytes upregulated expression of endothelial markers, fk-1(VEGFR2/KDR) and VE-cadherin [27]. Together, studies suggested that chemokines expression has critical role in VM formation in tumors.

Since GBM is hyper-vascular in nature, different drugs *e.g.* vatalanib, cediranib, sunitinib, etc. have been used against VEGF-VEGFR pathway to control abnormal angiogenesis in clinical trials [28-33]. Due to lower genetic instability in endothelial cells compared to tumor cells, it was anticipated that targeting VEGF-VEGFR pathways primarily in endothelial cells would decrease the tumor vasculature without imposing drug resistance. Regrettably, benefits of antiangiogenic treatments (AATs) are at best transitory, and this period of clinical benefit is followed by restoration of tumor growth [34].

In preclinical studies, VEGFR2 blockade in GBM through vatalanib, a receptor tyrosine kinase inhibitor, significantly increased tumor size as shown by DCE-MRI [35]. Vatalanib treatment induced hypoxia and was associated with the increased expression of VEGF, SDF-1, HIF-1alpha, VEGFR2, VEGFR3 and EGFR at the peripheral part of the tumors compared to that of central part of the treated rat glioma [36]. Activation of alternative pathways of angiogenesis, vasculogenesis and involvement of stem cells were observed following AATs in GBM [33,37,38], which are tightly regulated through bFGF, angiopoietin1/2, GCSF, and SDF1α [35]. It is possible that hypoxia accompanying AAT induces VM channel formation to enhance the tumor growth [39]. However, study involving Flk-1 gene knockdown or VEGFR2 kinase inhibitor SU1498 abrogated VEGFR2 activity and impaired vascular function by suppressed intracellular signaling cascades, including FAK and MAPK ERK1/2. Surprisingly, blockade of VEGF activity by the neutralizing antibody Bevacizumab (Avastin) failed to recapitulate the impact of SU1498, suggesting that VEGFR2 -mediated VM is independent of VEGF [11]. By considering the demand of AATs including avastin and vatalanib in GBM and other cancers clinical trials, VM phenomenon is required to investigate in depth.

Chemo-therapeutic options are limited in glioma. AATs (e.g. vatalanib) showed transient effect on glioma growth and resulted into aggressive phenotypes such as increased invasion and neovascularization. VM facilitates molecular and phenotypic reprograming of tumor cells into endothelial-like cells in GBM and other hyper-vascular tumors. AATs such as avastin treatment has been failed to control VM, which could be the reason of therapeutic resistance in some cancers GBM. Therefore, novel agents are required to target neovascularization (VM) in GBM tumors.
